# Molecular Mechanisms of Environmental Enrichment: Impairments in Akt/GSK3β, Neurotrophin-3 and CREB Signaling

**DOI:** 10.1371/journal.pone.0064460

**Published:** 2013-05-21

**Authors:** Yuan-Shih Hu, Nancy Long, Gustavo Pigino, Scott T. Brady, Orly Lazarov

**Affiliations:** Department of Anatomy and Cell Biology, University of Illinois at Chicago, Chicago, Illinois, United States of America; Nathan Kline Institute and New York University School of Medicine, United States of America

## Abstract

Experience of mice in a complex environment enhances neurogenesis and synaptic plasticity in the hippocampus of wild type and transgenic mice harboring familial Alzheimer's disease (FAD)-linked APPswe/PS1ΔE9. In FAD mice, this experience also reduces levels of tau hyperphosphorylation and oligomeric β-amyloid. Although environmental enrichment has significant effects on brain plasticity and neuropathology, the molecular mechanisms underlying these effects are unknown. Here we show that environmental enrichment upregulates the Akt pathway, leading to the downregulation of glycogen synthase kinase 3β (GSK3β), in wild type but not FAD mice. Several neurotrophic signaling pathways are activated in the hippocampus of both wild type and FAD mice, including brain derived neurotrophic factor (BDNF) and nerve growth factor (NGF), and this increase is accompanied by the upregulation of the BDNF receptor, tyrosine kinase B (TrkB). Interestingly, neurotrophin-3 (NT-3) is upregulated in the brains of wild type mice but not FAD mice, while insulin growth factor-1 (IGF-1) is upregulated exclusively in the brains of FAD mice. Upregulation of neurotrophins is accompanied by the increase of N-Methyl-D-aspartic acid (NMDA) receptors in the hippocampus following environmental enrichment. Most importantly, we observed a significant increase in levels of cAMP response element- binding (CREB) transcripts in the hippocampus of wild type and FAD mice following environmental enrichment. However, CREB phosphorylation, a critical step for the initiation of learning and memory-required gene transcription, takes place in the hippocampus of wild type but not of FAD mice. These results suggest that experience of wild type mice in a complex environmental upregulates critical signaling that play a major role in learning and memory in the hippocampus. However, in FAD mice, some of these pathways are impaired and cannot be rescued by environmental enrichment.

## Introduction

Environmental factors and lifestyle are well established as crucial contributing factors for the development of Alzheimer's disease (AD) [1]. We and others have shown that experience of transgenic mice expressing familial Alzheimer's disease (FAD)-linked mutant amyloid precursor protein (APP) and/or presenilin-1 (PS1) in environmental enrichment (EE) rescues impaired neurogenesis, enhances hippocampal long-term potentiation (LTP) and upregulates gene expression of molecular targets associated with learning and memory, synaptic plasticity and neuronal survival [2]–[4]. This suggests an overall increase of brain plasticity and synaptic function following experience of FAD mice in EE. In addition, environmental enrichment reduces pathological hallmarks in the brains of FAD mice. Specifically, level of soluble oligomeric Aβ, the neurotoxic precursor of amyloid plaques, is significantly reduced in the brains of FAD mice following EE [3]–[6]. Moreover, experience in EE decreased the level of hyperphosphorylated tau, the precursor of neurofibrillary tangles in brains of these mice [3], [7]. Interestingly, concomitant to reduced levels of hyperphosphorylated tau, we observed an upregulation of the main anterograde motor protein, kinesin-1, in the brain of enriched mice, suggesting that EE may enhance axonal transport [3].

In spite of the extensive use of EE and the high potential therapeutic value of this experimental paradigm for neurodegenerative diseases and aging, the molecular mechanisms underlying its effects are not fully elucidated. Several studies have reported that physical exercise activates phosphatidylinositol-3-kinase (PI3K)/Akt pathway in the skeletal muscles and in the hippocampus of wild type mice [8]–[10]. Akt, also known as Protein kinase B, is a major upstream modulator of glycogen synthase kinase 3 beta (GSK3β) in neurotrophin-dependent signaling pathways. Protein kinase B directly regulates GSK3β by phosphorylation of GSK3β at amino acid serine 9, thereby inactivating its kinase activity. Both GSK3β and cyclin-dependent kinase 5 (CDK5) are key kinases that phosphorylate tau *in vitro* and *in vivo* and their misregulation is implicated in the formation of neurofibrillary tangles [11]–[16]. Interestingly, both GSK3β and CDK5 are also key regulators of kinesin-based anterograde axonal transport [17], [18].

The predominant neurotrophic factor implicated in AD is brain derived neurotrophic factor (BDNF). Its activation of the tyrosine kinase B (TrkB) receptor modulates neuronal differentiation, neuronal survival and synaptic plasticity via multiple signaling pathways, i.e. extracellular signal regulated kinases (ERK) implicated in cell differentiation, PI3K/Akt implicated in cell survival, and phospholipase Cγ/protein kinase C (PLCγ/PKC) signaling pathways implicated in synaptic plasticity, respectively [19]. Brain derived neurotrophic factor-mediated TrkB activation can promote neuronal synaptic activity through activation of the transcription factor cyclic adenosine monophosphate (cAMP) response element-binding (CREB), a critical signal for the formation of long-term learning and memory [20]. Increased BDNF levels are also associated with enhanced activity of CREB, [21], which in turn drives the expression and activation of intracellular signaling pathways through the actions of two types of glutamate-gated ion channels; α-amino-3-hydroxy-5-methylisoxazole-4-propionic acid (AMPA) and N-methyl-D-aspartate (NMDA) receptors.

Upregulation of BDNF protein levels by physical exercise and environmental enrichment was shown previously by numerous studies, including ours [2], [22]–[25]. Alterations in BDNF levels are observed in the cortex and hippocampus of AD patients [26]–[29], and BDNF immunoreactivity is associated with senile plaques [30], [31]. Surprisingly, the nature of BDNF alteration in AD is controversial, with some reports suggesting that BDNF increases [32], and others suggesting it decreases in the hippocampus of AD patients [33], [34]. Interestingly, BDNF induces rapid dephosphorylation of tau protein via the PI3K/Akt signaling pathways [35]. Previously we demonstrated that BDNF gene expression is upregulated in APPswe/PS1ΔE9 mice following EE [2]. Nevertheless, several important questions remain unanswered. These include questions about the effect of EE on BDNF metabolism, the regulation of other neurotrophins and the activation of the pathway(s) downstream of TrkB activation that are directly mediated by EE.

The purpose of this study was to determine the signaling pathways underlying the effects of EE on the brains of nontransgenic and APPswe/PS1ΔE9 mice. Here we show that expression of the inactive form of GSK3β is upregulated in the brains of nontransgenic, but not in APPswe/PS1ΔE9 mice following EE. Similarly, levels of phosphorylated Akt are increased in the brains of nontransgenic, but not in APPswe/PS1ΔE9 mice following EE. In addition, the levels of several neurotrophic factors, such as BDNF and nerve growth factor (NGF) are also upregulated following EE, both of which are capable of activating Akt/GSK3β cascade. Experience in EE also increases the levels of neurotrophin-3 (NT-3) in the brains of nontransgenic but not APPswe/PS1ΔE9 mice, while insulin growth factor-1 (IGF-1) is only upregulated in the hippocampus of APPswe/PS1ΔE9 mice. Furthermore, we show that NMDA receptor 1 (NMDAR1), but not glutamate receptor-1 (GluR1), is upregulated following EE in the hippocampus of both nontransgenic and APPswe/PS1ΔE9 mice. Finally, we show that CREB transcription is upregulated following EE in the hippocampus of both nontransgenic and APPswe/PS1ΔE9 mice. However, CREB phosphorylation is upregulated only in the hippocampus of wild type mice, but not in the hippocampus of APPswe/PS1ΔE9 mice. This suggests that a critical step in learning and in the formation of long-term memories is defective in APPswe/PS1ΔE9, and cannot be rescued by EE. This study provides novel insights on a network of cascades altered by EE in the brains of wild type and APPswe/PS1ΔE9 mice. These findings provide new information about the inhibitory effect of APPswe/PS1ΔE9 on stimulus-induced upregulation of the Akt/GSK3β and NT-3 pathways, which may underlie or contribute to defective CREB signaling. Defective EE-induced CREB phosphorylation APPswe/PS1ΔE9 mice may suggest that additional intervention will be required for a complete rescue of learning and memory impairments in AD.

## Materials and Methods

### Transgenic animals

**Ethics Statement:** All animal procedures were approved by the University of Illinois at Chicago Institutional Animal Care and Use Committee (IACUC). FAD-linked APPswe/PS1ΔE9 transgenic mice coexpressing human PS1 encoding ΔE9 mutation, and mouse APP containing humanized Aβ and the Swedish mutation (K595N, M596L) were generated as previously described [36]. Transgenic mice and nontransgenic littermates were maintained in standard laboratory conditions (14/10 hr light-dark cycle) and with full access to food and water *ad libitum*. For brain tissue collections, animals were euthanized with isoflurane followed by cervical dislocation. Brain tissues were quickly dissected into different regions (e.g. cortex, hippocampus etc.) and frozen immediately in liquid nitrogen. All samples were stored in −80°C until analyzed.

### Environmental enrichment

Twenty one day-old male APPswe/PS1ΔE9 mice (N = 14, N = 7 for protein analysis, N = 7 for mRNA analysis) and their nontransgenic littermates (N = 14, N = 7 for protein analysis, N = 7 for mRNA analysis) either experienced environmental enrichment for a period of 1 month or were maintained in standard housing (SH) conditions as described previously [3]. Mice were maintained in groups of 3–5 males/cage. The enriched environment was composed of running wheels, color tunnels, visually stimulating toys, and free access to food and water in the enlarged cages (approximately 24×17×11 inches in dimensions). Objects in the cage were changed and repositioned for novel stimulation every day. Mice were exposed to environmental enrichment for 3 hours everyday and returned to the standard housing cage (approximately 11×6×8 inches in dimensions) for the rest of the day. Control groups of APPswe/PS1ΔE9 mice (N = 14, N = 7 for protein analysis, N = 7 for mRNA analysis) and their nontransgenic littermates (N = 14, N = 7 for protein analysis, N = 7 for mRNA analysis) were singly housed in standard laboratory conditions for 1 month. For the study comparing the effects of young versus old mice, male APPswe/PS1ΔE9 mice (N = 5) and their nontransgenic littermates (N = 5) were housed in standard laboratory condition for either 2 or 6 months. The number of animals used for each experiment is indicated in the result sections and in each individual figure legends.

### SDS-PAGE and Western blot Analysis

Hippocampal and cortical protein extraction were prepared in ROLB buffer as described before [37]. Briefly, 30 µg of detergent soluble protein samples were separated on 7.5% acrylamide gels and transferred onto 0.45 µm nitrocellulose membrane (Bio-Rad) for 2 hours at 100 mV in transfer buffer (25 mM Tris, 192 mM glycine, pH 8.3). Membrane blots were blocked for 2 hours in blocking solution (1% BSA in TBS) at room temperature, followed by incubation of primary antibodies diluted in blocking solution overnight at 4°C. On the next day, the membranes were washed three times with TBST (0.1% Tween-20), and incubated in secondary antibody diluted in TBST for 1 hour. Membranes were visualized with ECL™ Plus chemiluminescent substrate (GE Healthcare) and protein expression levels were quantified by densitometric analysis using ImageJ1.41o software (National Institutes of Health, Bethesda, MD, USA). Primary antibodies used in this study were polyclonal rabbit anti-phosphorylated GSK3β ser 9 (1∶2000, Cell Signaling), monoclonal mouse anti-GSK3β (1∶2500, BD transduction), polyclonal rabbit anti-Akt (1∶2000, Cell Signaling), polyclonal rabbit-anti-phosphorylated Akt Ser 437 (1∶2000, Cell Signaling), polyclonal rabbit anti-ERK C-16 (1∶1000, Santa Cruz), monoclonal mouse anti-phosphorylated ERK E-4 (1∶1000, Santa Cruz), monoclonal mouse anti-CREB (1∶1000, Cell Signaling), monoclonal mouse anti-phosphorylated CREB ser133 (1∶1000, Cell Signaling), and monoclonal mouse anti-actin (1∶2500, Millipore). Secondary horse peroxidase antibodies used in this study were rabbit anti-mouse HRP (1∶5000, Pierce) and donkey anti-rabbit HRP (1∶20,000, Promega).

### BDNF ELISA Assay

The expression level of BDNF was measured using an ELISA kit, BDNF E_max_ ImmunoAssay System (Promega, WI, USA), according to manufacturer's instructions. Brain tissues were homogenized in modified protein extraction buffer as described [38], followed by BCA quantification assay to determine protein concentration. To measure BDNF levels, 96-well immunoplates were coated with 100 µL per well of monoclonal anti-mouse-BDNF antibody (1∶2000). After an overnight incubation at 4°C, plates were washed three times with wash buffer and the protein samples (100 µL) were incubated in coated wells for 2 hours at room temperature. Immobilized antigen was incubated with an anti-human BDNF antibody for 2 hours at room temperature. The plates were then incubated with an anti-IgY HRP for 1 hour at room temperature followed by TMB/peroxidase substrate solution and 1 M HCl (100 µL/well). The colorimetric reaction product was measured at 450 nm using a microplate reader. BDNF concentration was determined based on linear regression of the BDNF standards (range = 7.8–500 pg/mL purified mouse BDNF) that were incubated under similar conditions in each assay. The sensitivity of the assay is about 15 pg/g of BDNF, and cross-reactivity with other related neurotrophic factors (Nerve growth factor, Neurotrophin-3 and Neurotrophin-4) is less than 3%. All samples were assayed in duplicate.

### RNA extraction

Total RNA was isolated from brain tissue using RNAeasy Mini Kit (Qiagen), according to manufacturer's instruction. Briefly, about 20 mg of tissue were manually homogenized in 500 µL of buffer RLT extraction buffer. The homogenate was centrifuged for 3 minutes at full speed, and the supernatant was transferred to a new tube. A second centrifugation was performed to remove any remaining cellular debris, followed by adding 95% ethanol for RNA precipitation and binding onto the column. The column was centrifuged and washed several times, and the RNA was eluted from the column using RNase-free water. Total RNA concentration was determined using NanoDrop® spectrophotometer (NanoDrop Technologies, Wilmington, DE, USA) at 260 nm/280 nm, and RNA integrity was determined by running a 1% denaturing agarose gel electrophoresis. Total RNA samples were stored at −80°C until further analysis.

### Reverse Transcription and quantitative Polymerase Chain Reaction (qPCR)

Complementary DNA (cDNA) synthesis was performed with SuperScript®III First-strand synthesis SuperMix (Invitrogen), using 1 µg of total RNA and oligo dT primers. cDNA was further diluted in deionized water and stored at −20°C. The lists of primer sequences and references are listed in Table S1. Primer efficiency was tested using conventional PCR. Cycling conditions were: 10 min at 95°C, followed by 40 cycles at 95°C for 30 sec, 60°C for 30 sec and 72°C for 30 sec. Samples were analyzed in triplicate and a melting curve analysis was performed in each sample at the end of qPCR reaction. Expression level of each gene was determined by BioRad iQ5 icycler real time PCR system employing iQ SYBR Green Supermix (BioRad). Expression levels of 18S ribosomal RNA (18S rRNA) and Glyceraldehyde 3-phosphate dehydrogenase (GAPDH) were used as internal controls. Relative gene expression was determined by 2^−ΔΔCt^ method [39]. The threshold cycle (Ct) value was determined for target genes and the endogenous internal controls in each sample. The difference between target gene Ct and internal control Ct was determined for each sample, resulting in the ΔCt value. The ΔCt of a calibrator sample was subtracted from each sample ΔCt to yield the ΔΔCt value. Relative fold change was calculated as 2^−ΔΔCt^.

### Statistical Analysis

Data are presented as mean ± SE. All statistical analyses were performed using GraphPad Prism version 5.00 for Windows (GraphPad Software, San Diego California USA). All biochemical analyses (ELISA, densitometry, RT-PCR data) were analyzed using Student's t test or one-way ANOVA, followed by Tukey's post hoc test. All results were considered statistically significant when *P*<0.05.

## Results

### Environmental enrichment downregulates kinase activities involved in pathological tau phosphorylation

**Figure 1:**
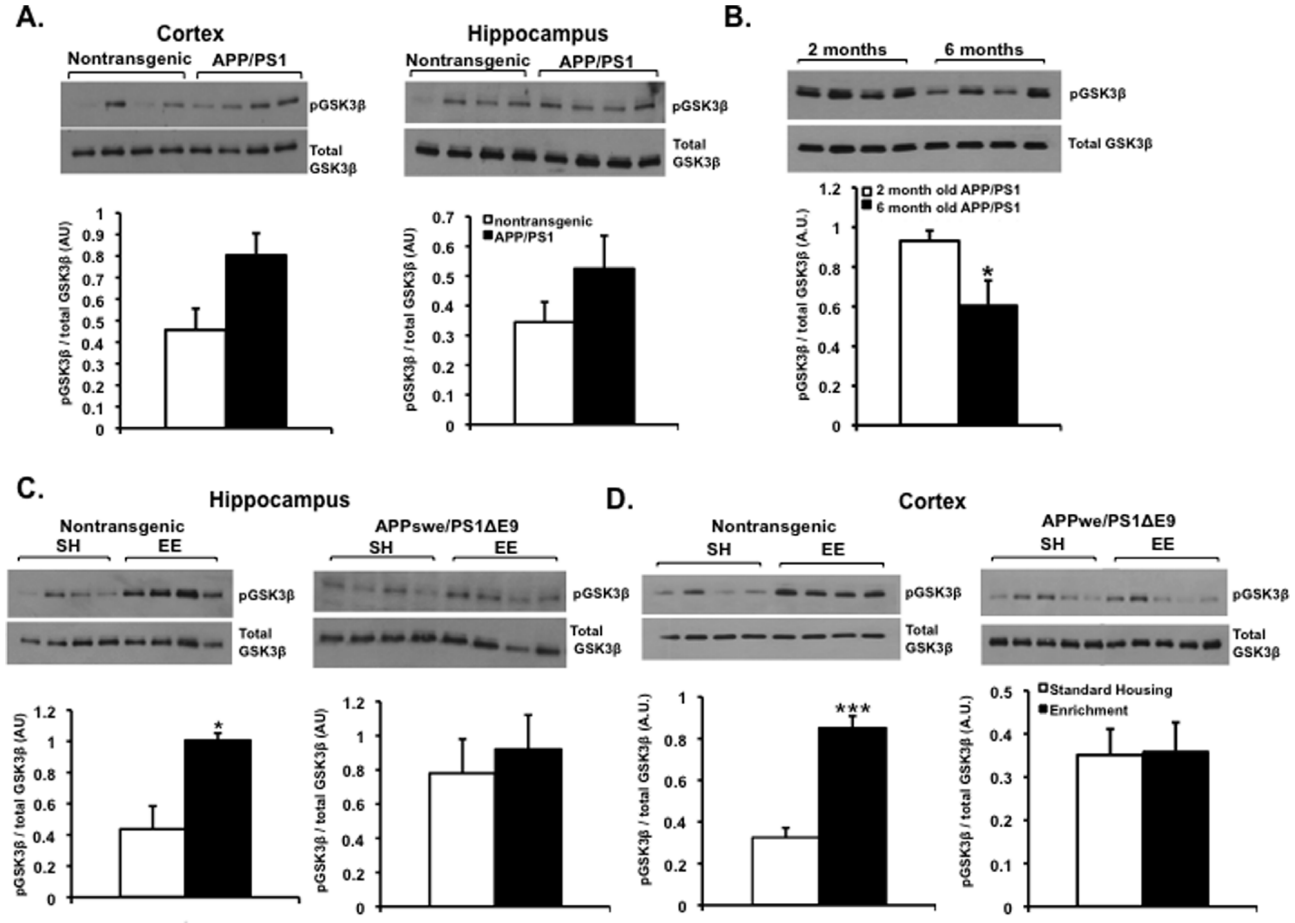
Expression of inactive GSK3β is upregulated following environmental enrichment in the brains of nontransgenic but not APPswe/PS1ΔE9 mice. (A) Expression levels of GSK3β and its inactive form pGSK3β ser 9 are comparable in the cortex and hippocampus of nontransgenic and APPswe/PS1ΔE9 mice at 2 months of age as detected by Western blot analysis and densitometric quantification (*P* = 0.2419 cortex, *P* = 0.2161 hippocampus, N = 4 for nontransgenic, N = 4 for APPswe/PS1ΔE9, Student's t test). (B) Levels of pGSK3β ser 9 decrease in the hippocampus of 6 month-old APPswe/PS1ΔE9 (N = 4) compared to levels at 2 months of age (N = 4), suggesting an increased activity of GSK3β at 6 months (*P<0.05, Student's t test) as total GSK3β levels are preserved. (C,D) Levels of pGSK3β ser 9 increase in the hippocampus (C) and cortex (D) of nontransgenic but not of APPswe/PS1ΔE9 mice following experience in an enriched environment. Values are means ± SE (arbitrary units) [*P<0.05 nontransgenic hippocampus (N = 4 for SH, N = 4 for EE), *P* = 0.4685 APPswe/PS1ΔE9 hippocampus (N = 4 for SH, N = 4 for EE), ***P<0.0001 nontransgenic cortex (N = 4 for SH, N = 4 for EE), *P* = 0.9302 APPswe/PS1ΔE9 cortex (N = 5 for SH, N = 5 for EE), Student's t test].

Activities of tau and kinesin are both tightly regulated by the level of phosphorylation. Intrigued by our previous observation that EE attenuates tau phosphorylation and upregulates kinesin-1 levels, we examined the role of kinase activities implicated in the regulation of tau and kinesin phosphorylation. Numerous studies in FAD mouse models [16], [40]–[42] and in humans [43]–[47] suggest that misregulated GSK3β activity plays a critical role in the pathogenesis of AD. One possibility is that GSK3β activity is compromised while its level of expression is unaltered. To evaluate this, we used an antibody that recognizes phosphorylated GSK3β at serine 9 (pGSK3β ser 9), which corresponds to the inactive form of GSK3β [48]. To determine whether GSK3β signaling pathway is compromised in FAD, we compared the ratio of pGSK3β ser 9 to total GSK3β expression levels in APPswe/PS1ΔE9 and wild type littermates maintained in standard housing conditions by Western blot analysis. Although the trend was an increased ratio of inactive GSK3β to total GSK3β, the differences were not statistically significant in cortex and hippocampus of 2 month-old wild type mice (N = 4) compared to APPswe/PS1ΔE9 (N = 4) (Figure 1A), suggesting that in young APPswe/PS1ΔE9 mice, GSK3β levels and activity are not altered. To test whether changes in GSK3β levels were age-dependent, we examined the expression and activity levels of GSK3β in hippocampal protein extracts of APPswe/PS1ΔE9 mice at 2 and 6 months of age, corresponding to pre- and post-onset of amyloid deposition, respectively (N = 4 for 2 month-old, N = 4 for 6 month-old). The result showed a significant decrease in pGSK3β ser 9/total GSK3β levels in 6 month-old mice, suggesting an increased activity of GSK3β at 6 months compared to 2 months of age (Figure 1B).

To examine whether EE regulates the level of pGSK3β ser 9, and hence GSK3β kinase activity, we examined the protein expression levels of pGSK3β ser 9 and total GSK3β in the cortex and hippocampus of nontransgenic and APPswe/PS1ΔE9 mice following experience in an enriched environment by Western blot analysis. Levels of pGSK3β ser 9 were significantly upregulated in both cortex and hippocampus of nontransgenic mice following EE [N = 4 for SH and N = 4 for EE, (Figure 1C, D left panels)], suggesting a significant reduction in GSK3β activity in the brains of these mice following EE. However, this effect was not observed in APPswe/PS1ΔE9 mice following EE [N = 4 for SH and N = 5 for EE, (Figure 1C,D right panels)], suggesting that expression of APPswe/PS1ΔE9 compromises EE-induced downregulation of pGSK3β activity.

### Reduced GSK3β activity in nontransgenic mice may be induced by the activation of its upstream PI3K/Akt signaling pathway

GSK3β is one of the major downstream substrates of Akt in the PI3K/Akt-dependent signaling pathway.

**Figure 2:**
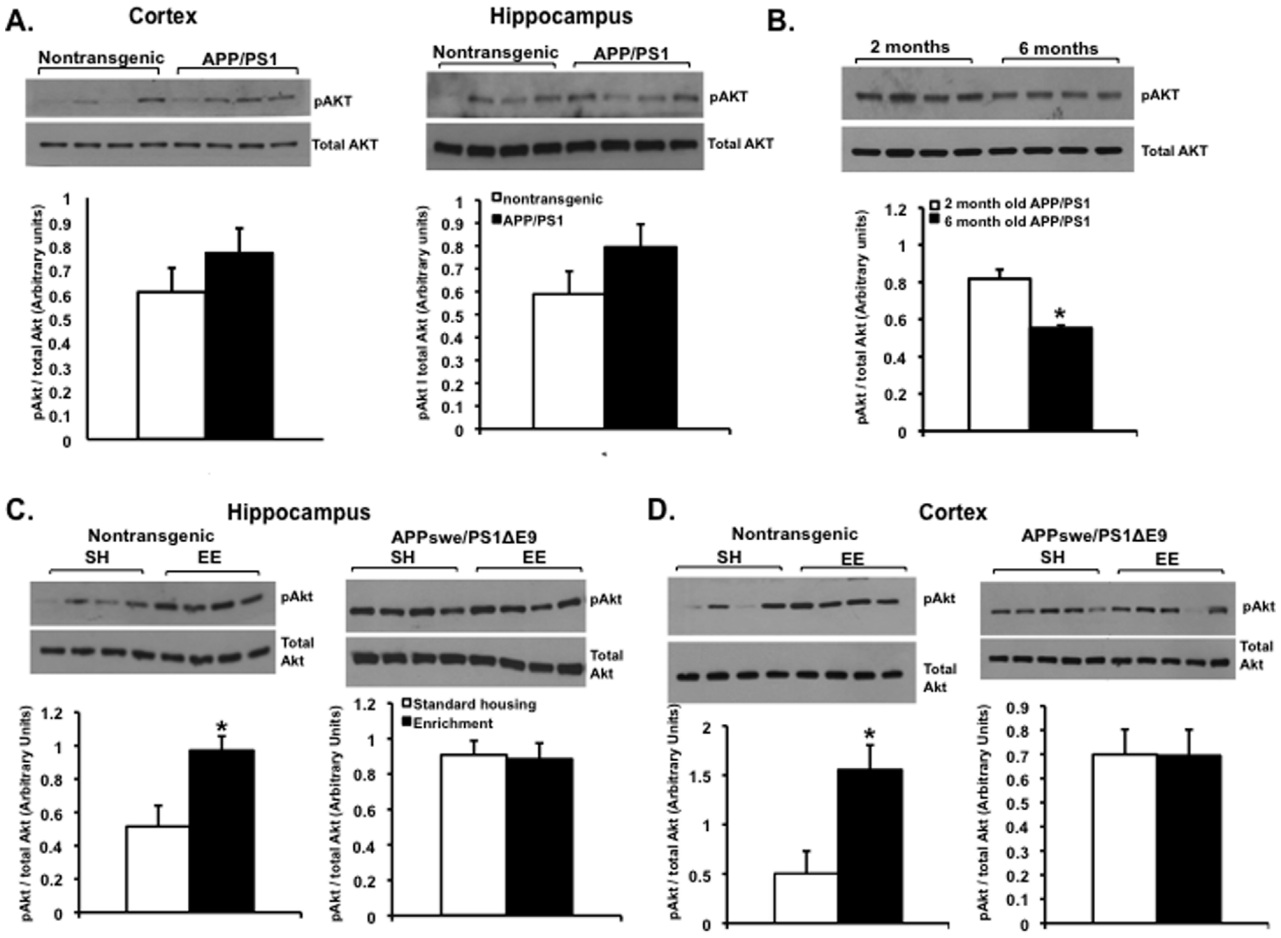
Akt expression and activity are upregulated following environmental enrichment in the brains of nontransgenic but not APPswe/PS1ΔE9 mice. (A) Expression levels of the active form of Akt kinase, pAkt ser 437, is comparable in the cortex and hippocampus of nontransgenic and APPswe/PS1ΔE9 mice at 2 months of age as detected by Western blot analysis and densitometric quantification (*P* = 0.4331 cortex, *P* = 0.4740 hippocampus, N = 4 for nontransgenic, N = 4 for APPswe/PS1ΔE9, Student's t test). (B) Levels of pAkt ser 437 decrease in the hippocampus of 6 month-old APPswe/PS1ΔE9 mice (N = 4) compared to levels at 2 months of age (N = 4), suggesting decreased activity at 6 months (*P<0.05, Student's t test). (C,D) Levels of pAkt ser 437 increase in the hippocampus (C) and cortex (D) of nontransgenic but not of APPswe/PS1ΔE9 mice following experience in an enriched environment, again with little or no change in total Akt levels. Values are means ± SE [(arbitrary units) *P<0.05 nontransgenic hippocampus (N = 4 for SH, N = 4 for EE), *P* = 0.9856 APPswe/PS1ΔE9 hippocampus (N = 4 for SH, N = 4 for EE), *P<0.05 nontransgenic cortex (N = 4 for SH, N = 4 for EE), *P* = 0.8602 APPswe/PS1ΔE9 cortex (N = 5 for SH, N = 5 for EE), Student's t test].

Like GSK3β, the activity of Akt is also tightly regulated by phosphorylation by its upstream regulator, phosphoinositol-3 kinase (PI3K). However, unlike GSK3β, phosphorylation of Akt at serine 437 by PI3K increases its kinase activity. Thus, we examined the expression levels of phosphorylated Akt using antibodies that recognize phosphorylation of Akt at Serine 437 (pAkt ser 437) and total Akt in the cortex and hippocampus of these mice. As in the case of GSK3β, we found no significant difference in the level of total Akt or pAkt ser 437 at 2 months of age in APPswe/PS1ΔE9 mice (N = 4) compared to their nontransgenic littermates [N = 4, (Figure 2A)]. However, we observed a significant decrease of pAkt ser 437 level in 6 month-old mice compared to 2 month-old mice [N = 4 for 2 month-old, N = 4 for 6 month-old, (Figure 2B)]. This may suggest that upregulation of GSK3β activity at 6 months of age in the brains of APPswe/PS1ΔE9 may directly result from Akt downregulation.

Several studies have reported that physical exercise activates the PI3K/Akt survival-promoting pathway, which in turn regulates GSK3β activity. This effect is thought to be mediated by increased levels of neurotrophic factors [8], [9]. Based on our observation of reduced GSK3β activity following EE in nontransgenic mice, we analyzed protein expression levels of pAkt Ser 437 in the cortex and hippocampus of standard housing and enriched mice (N = 5 per group) by Western blot analysis. We observed that pAkt Ser 437 expression level was upregulated in the cortex and hippocampus of nontransgenic mice that experienced EE (Figure 2C,D left panels), suggesting increased Akt activity following EE. In agreement with our earlier findings on GSK3β, Akt activity in cortex and hippocampus of APPswe/PS1ΔE9 mice was unaffected by EE (Figure 2C,D left panels). Taken together, this suggests that EE-induced Akt/GSK3β signaling may be defective in APPswe/PS1ΔE9 mice.

### Expression levels of BDNF are comparable in APPswe/PS1ΔE9 and nontransgenic littermates at 2 months of age

The binding of brain derived neurotrophic factor (BDNF) to its receptor activates the PI3K/Akt signaling pathway [49]. Changes in BDNF expression were observed in AD patients [26] and BDNF immunoreactivity was closely associated with senile plaques [30], [31]. However, whether the levels of BDNF and its receptors are increased [32], [50], or decreased is still controversial in both human post-mortem Alzheimer's brains and in many AD mouse models. [33], [34], [51]. In fact, depending on the regions studied, the levels of different neurotrophins in AD patients may vary significantly [29], [34].

**Figure 3:**
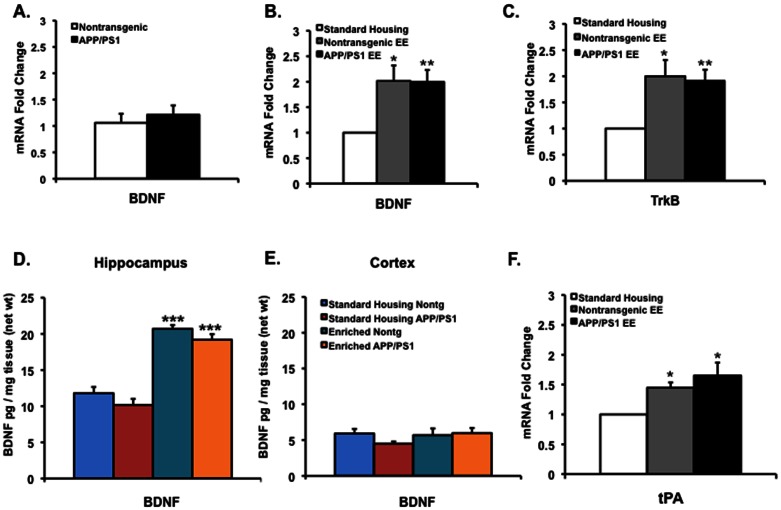
Hippocampus-specific upregulation of BDNF following environmental enrichment in nontransgenic and APPswe/PS1ΔE9 is accompanied by increased levels of TrkB receptors and tPA. (A) Real time RT-PCR of RNA extract of the hippocampus of nontransgenic and APPswe/PS1ΔE9 mice revealed comparable levels of BDNF in the hippocampus of nontransgenic and APPswe/PS1ΔE9 at 2 months of age (*P* = 0.5517, N = 5 for nontransgenic SH, N = 5 for APPswe/PS1ΔE9 SH, Student's t test). (B) BDNF levels increase in the hippocampus of nontransgenic and APPswe/PS1ΔE9 mice following environmental enrichment as detected by real time RT-PCR [*P<0.05 for nontrangenic SH (N = 5) vs. nontransgenic EE (N = 5), **P<0.01 for APPswe/PS1ΔE9 SH (N = 5) vs. APPswe/PS1ΔE9 EE (N = 5), Student's t test]. (C) Upregulation of TrkB receptors was also observed in the hippocampus of nontransgenic and APPswe/PS1ΔE9 mice following environmental enrichment as detected by real time RT-PCR [*P<0.05 for nontrangenic SH (N = 5) vs. nontransgenic EE (N = 5), **P<0.01, for APPswe/PS1ΔE9 SH (N = 5) vs. APPswe/PS1ΔE9 EE (N = 7), Student's t test]. (D,E) BDNF levels increase in the hippocampus [***P<0.001 for nontransgenic SH (N = 6) vs. nontransgenic EE (N = 6), ***P<0.001 for APPswe/PS1ΔE9 SH (N = 6) vs. APPswe/PS1ΔE9 EE (N = 6), one-way ANOVA], but not in the cortex [*P* = 0.8356 for nontransgenic SH (N = 6) vs. nontransgenic EE (N = 6), *P* = 0.0842 for APPswe/PS1ΔE9 SH (N = 6) vs. APPswe/PS1ΔE9 EE (N = 6), one-way ANOVA] of nontransgenic and APPswe/PS1ΔE9 following environmental enrichment as determined by ELISA. (F) Upregulation of tPA mRNA level in the hippocampus of nontransgenic and APPswe/PS1ΔE9 following environmental enrichment as determined by real time RT-PCR [*P<0.05 for nontrangenic SH (N = 6) vs. nontransgenic EE (N = 6), *P<0.05, for APPswe/PS1ΔE9 SH (N = 6) vs. APPswe/PS1ΔE9 EE (N = 7)].

To address this, we first asked whether mRNA levels of BDNF are altered early in life in APPswe/PS1ΔE9 mice. For this purpose, levels of BDNF mRNA were examined in the hippocampus of nontransgenic and APPswe/PS1ΔE9 mice at 2 months of age, before the onset of AD pathology. While many studies have shown altered expression levels of BDNF in AD patients and AD mouse models at older ages, we did not observe any significant difference in the mRNA expression level of BDNF at 2 months of age, raising the possibility that alterations in BDNF mRNA levels during progression of AD pathology may be through indirect mechanisms rather than by the direct effects of APPswe/PS1ΔE9 mutant expression, or that alterations in BDNF expression are pronounced at a later stage (Figure 3A).

### Environmental enrichment upregulates BDNF and TrkB receptor expression exclusively in the hippocampus of nontransgenic and APPswe/PS1ΔE9 mice

To determine whether environmental enrichment upregulates BDNF at 2 months of age, mRNA expression levels of BDNF were examined in the hippocampus of nontransgenic and APPswe/PS1ΔE9 mice following EE by real time RT-PCR. We observed a two-fold increase in BDNF mRNA expression level in the hippocampus of enriched mice compared to their standard housing littermates in both nontransgenic and APPswe/PS1ΔE9 mice (Figure 3B), suggesting that APPswe/PS1ΔE9 mice retain the ability to upregulate BDNF to the same extent as nontransgenic mice. To gain more insight into the downstream effectors of BDNF modulated by EE, we sought to examine whether EE-induced upregulation of BDNF mRNA is accompanied by upregulation of its receptor TrkB. Here we show that TrkB mRNA level was significantly upregulated following EE in both nontransgenic and APPswe/PS1ΔE9 mice (Figure 3C).

To confirm the upregulation of BDNF protein in the hippocampus of nontransgenic and APPswe/PS1ΔE9 mice following EE, and to examine whether this upregulation is confined to the hippocampus, we examined protein expression levels of BDNF in cortex and hippocampus of nontransgenic and APPswe/PS1ΔE9 mice by ELISA. We found a marked increase in BDNF in the hippocampus of both nontransgenic and APPswe/PS1ΔE9 mice following EE (Figure 3D). However, there was no change in BDNF levels in the cortex of these mice (Figure 3E).

Environmental enrichment-induced BDNF increases may be due to either increased production of BDNF or increased processing of immature proBDNF into mature BDNF. Interestingly, studies have suggested that physical exercise affects hippocampal plasticity by promoting proBDNF proteolytic cleavage, thereby increasing the levels of mature BDNF [52], [53]. To address the possibility that EE-induced increases in BDNF were due to its enhanced proteolytic cleavage, we examined the expression of tissue-type plasminogen activator (tPA), the enzyme that converts plasminogen into plasmin, which in turn cleaves proBDNF to yield mature BDNF. Interestingly, we observed a significant upregulation of tPA mRNA expression following EE in both nontransgenic and APPswe/PS1ΔE9 mice, suggesting that proteolytic processing of proBDNF to mature BDNF may be enhanced following EE (Figure 3F).

**Figure 4:**
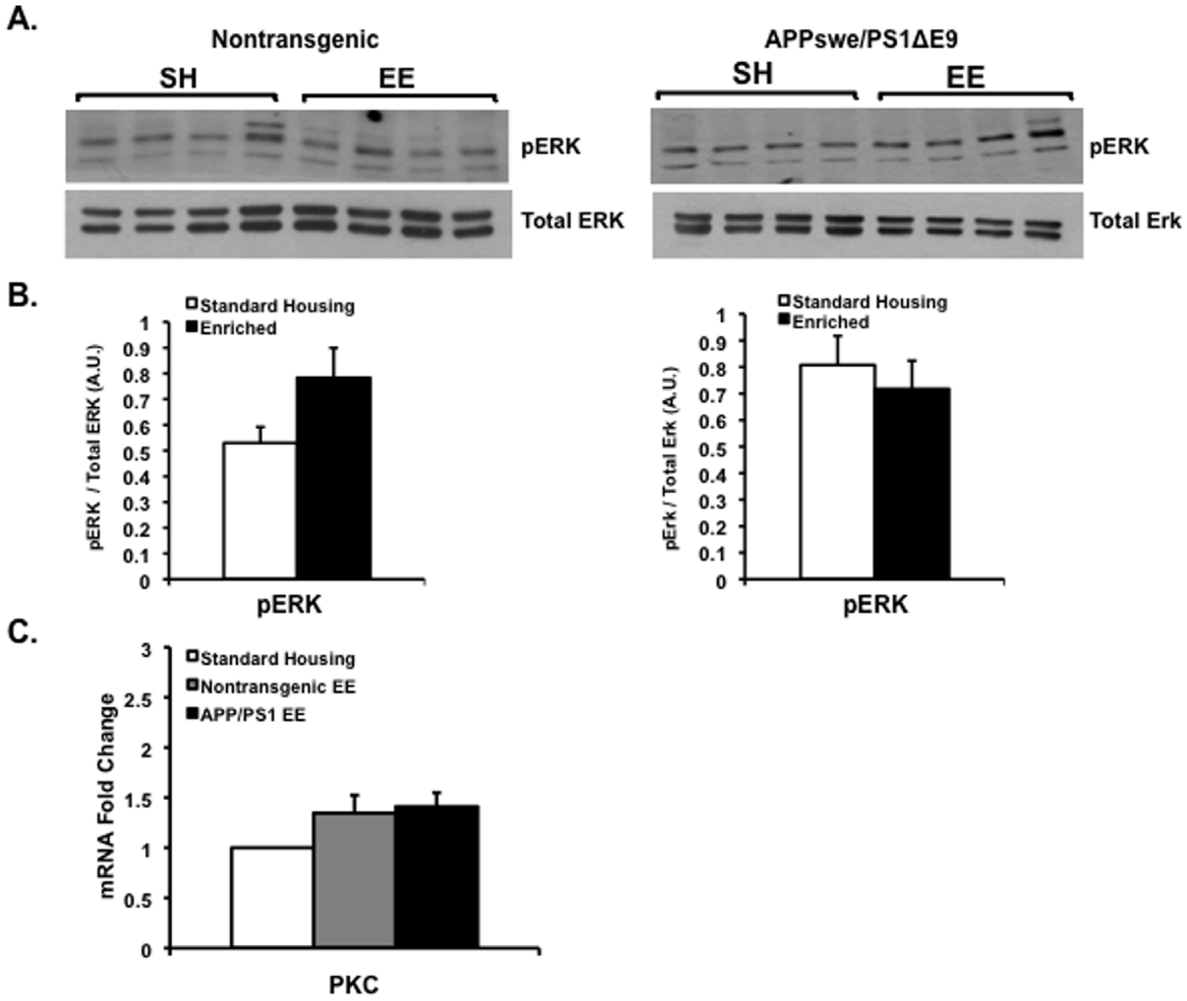
No changes in levels of Akt-induced ERK or PKC signaling following environmental enrichment. (A,B) Protein expression levels of pERK were comparable in the cortex of nontransgenic and APPswe/PS1ΔE9 mice following experience in an enriched environment as detected by Western blot analysis (A) and densitometric quantification (B), [*P* = 0.5874 for nontransgenic (N = 4 per group), *P* = 0.1031 for APPswe/PS1ΔE9 (N = 4 per group), Student's t test]. (C) No statistically significant change in the level of PKC following environmental enrichment as detected by real-time RT-PCR [*P* = 0.6504 for nontransgenic SH (N = 6) vs. nontransgenic EE (N = 6), *P* = 0.1364 for APPswe/PS1ΔE9 SH (N = 6) vs. APPswe/PS1ΔE9 EE (N = 6), Student's t test].

In addition to the PI3K/Akt pathway, BDNF-TrkB signaling modulates neuronal synaptic plasticity via extracellular signal regulated kinases (ERK), and the PLCγ/PKC signaling pathways. To address whether experience of wild type and transgenic mice in EE upregulates these pathways, we examined levels of total ERK and phosphorylated ERK (pERK) in the cortex of enriched nontransgenic and APPswe/PS1ΔE9 mice by Western blot analysis. While in wild type mice there was a trend of increased levels of pERK following EE, we observed no statistically significant changes in levels of either total ERK or pERK (Figure 4A,B). Gene expression level of PKCγ mRNA by real time RT-PCR revealed no change in PKCγ expression following enrichment in either genotype (Figure 4C).

### EE upregulates NGF expression and differentially modulates levels of NT-3 and IGF-1 in the hippocampus of nontransgenic and APPswe/PS1ΔE9 mice

**Figure 5:**
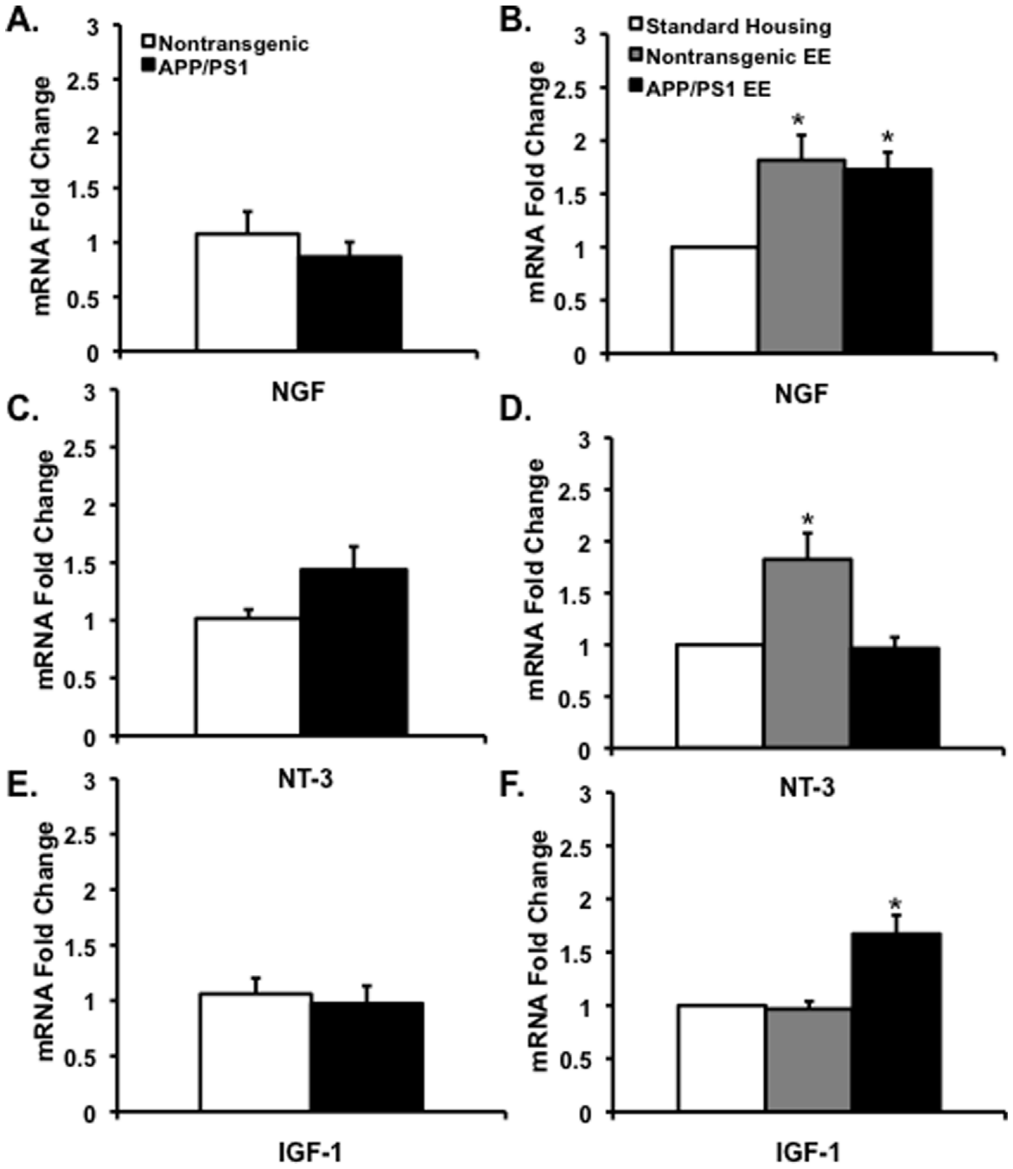
Differential regulation of neurotrophins following environmental enrichment in the brains of nontransgenic and APPswe/PS1ΔE9 mice. (A,C,E) mRNA levels of NGF (A), NT-3 (B) and IGF-1 (C) are comparable in the hippocampus of nontransgenic and APPswe/PS1ΔE9 mice at 2 months of age, as determined by real time-RT-PCR (NGF, *P* = 0.3945; NT-3, *P* = 0.0914 and IGF-1, *P* = 0.6949, N = 6 for nontransgenic, N = 6 for APPswe/PS1ΔE9, Student's t test). (B) NGF levels increased in the hippocampus of nontransgenic and APPswe/PS1ΔE9 mice following environmental enrichment [*P<0.05 for nontransgenic SH (N = 6) vs. nontransgenic EE (N = 6), *P<0.05 for APPswe/PS1ΔE9 SH (N = 6) vs. APPswe/PS1ΔE9 EE (N = 6), Student's t test]. (D) Levels of NT-3 were upregulated in the hippocampus of nontransgenic but not APPswe/PS1ΔE9 following environmental enrichment [*P<0.05 for nontransgenic SH (N = 6) vs. nontransgenic EE (N = 6), *P* = 0.0640 for APPswe/PS1ΔE9 SH (N = 6) vs. APPswe/PS1ΔE9 EE (N = 7), Student's t test]. (E) Levels of IGF-1 increased following environmental enrichment in the hippocampus of APPswe/PS1ΔE9 but not nontransgenic mice [P = 0.4077 for nontransgenic SH (N = 7) vs. nontransgenic EE (N = 7) *P<0.05 for APPswe/PS1ΔE9 SH (N = 6) vs. APPswe/PS1ΔE9 EE (N = 6), Student's t test].

To determine whether other neurotrophic factors may be upregulated in the hippocampus of nontransgenic and APPswe/PS1ΔE9 mice, we examined levels of the nerve growth factor (NGF), neurotrophin-3 (NT-3) and insulin growth factor-1 (IGF-1), that were previously reported to be upregulated in the brains of adult rats following EE [24], [54]–[58]. These neurotrophins have been shown to be differentially regulated in the brains of the *APP23* transgenic mouse model [4]. Using real time RT-PCR we observed that none of the neurotrophic factors were deficient in the hippocampus of 2 month-old APPswe/PS1ΔE9 mice, when compared to their nontransgenic littermates (Figure 5A, C, E). However, NGF levels were upregulated in the hippocampi of both nontransgenic and APPswe/PS1ΔE9 mice following EE (Figure 5B). Interestingly, we observed a significant increase in NT-3 mRNA expression in the hippocampus of nontransgenic mice that experienced EE, but not in enriched APPswe/PS1ΔE9 mice (Figure 5D). Conversely, IGF-1 mRNA expression was upregulated in the hippocampus of enriched APPswe/PS1ΔE9 mice, but not in the hippocampus of enriched nontransgenic mice (Figure 5F). This suggests that EE exhibits differential effects in the hippocampus of nontransgenic and APPswe/PS1ΔE9 mice. Another possibility is that expression of mutant APPswe/PS1ΔE9 induces a blockage of EE-induced NT-3, while the upregulation of IGF-1 is a compensatory mechanism for neurotrophin blockage. Nevertheless, further experiments are warranted in order to unravel the mechanism underling these differences.

### Learning and memory-linked CREB phosphorylation is enhanced in wild type but not APPswe/PS1ΔE9 mice following EE

**Figure 6:**
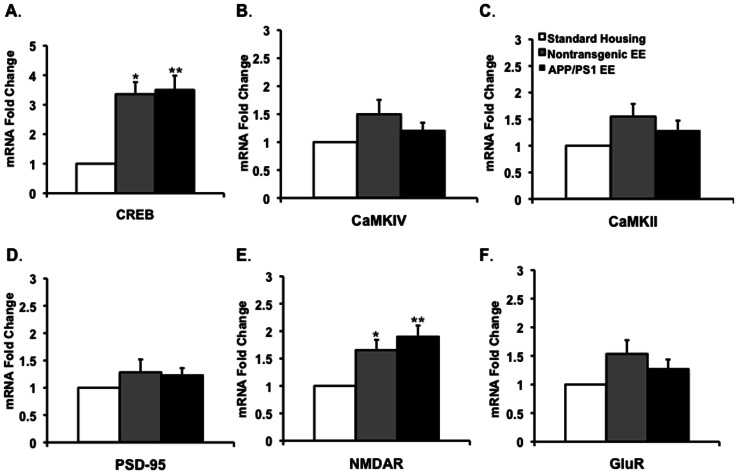
Upregulation of CREB and NMDAR transcription in the hippocampus of nontransgenic and APPswe/PS1ΔE9 mice following environmental enrichment. Real time RT-PCR suggests that levels of (A) CREB were significantly upregulated in the hippocampus of nontransgenic and APPswe/PS1ΔE9 mice following environmental enrichment [*P<0.05 for nontransgenic SH (N = 7) vs. nontransgenic EE (N = 7), **P<0.01 for APPswe/PS1ΔE9 SH (N = 6) vs. APPswe/PS1ΔE9 EE (N = 6), Student's t test], but not levels of (B) CaMKIV [*P* = 0.2326 for nontransgenic SH (N = 6) vs. nontransgenic EE (N = 6), *P* = 0.5772 for APPswe/PS1ΔE9 SH (N = 6) vs. APPswe/PS1ΔE9 EE (N = 7)], (C) CaMKII [*P* = 0.0642 for nontransgenic SH (N = 7) vs. nontransgenic EE (N = 7), *P* = 0.3524 for APPswe/PS1ΔE9 SH (N = 6) vs. APPswe/PS1ΔE9 EE (N = 7)] or (D) PSD-95 [*P* = 0.4337 for nontransgenic SH (N = 7) vs. nontransgenic EE (N = 7), *P* = 0.4236 for APPswe/PS1ΔE9 SH (N = 7) vs. APPswe/PS1ΔE9 EE (N = 7)]. (E,F) Levels of NMDAR1 [*P<0.05 for nontransgenic SH (N = 6) vs. nontransgenic EE (N = 6), **P<0.01 for APPswe/PS1ΔE9 SH (N = 6) vs. APPswe/PS1ΔE9 EE (N = 6)], but not GluR [*P* = 0.1256 for nontransgenic SH (N = 6) vs. nontransgenic EE (N = 6), *P* = 0.3937 for APPswe/PS1ΔE9 SH (N = 6) vs. APPswe/PS1ΔE9 EE (N = 6)], are upregulated in the hippocampus following environmental enrichment.

Expression of cAMP response element-binding (CREB) is critical for formation of long-term memory and learning [20]. Brain derived neurotrophic factor-mediated TrkB activation has been shown to promote neuronal synaptic activity through activation of the transcription factor, CREB, which drives the expression and activation of intracellular signaling pathways through the action of two types of glutamate-gated ion channels; AMPA and NMDA receptors [20], [59]. To test the hypothesis that EE upregulates learning and memory-dependent signals, we examined gene expression of CREB following EE. We show that EE upregulated mRNA expression level of CREB by 3.5 fold (Figure 6A), possibly through the activation of neurotrophins and growth factors.

Calcium-calmodulin-dependent protein kinase IV subunit (CaMKIV) has emerged as the most important Ca^2+^-activated CREB kinase *in vivo* [60], [61]. Therefore we examined the expression of CaMKIV in the hippocampus of EE mice. CaMKIV acts as a kinase for the CREB binding protein (CBP). CREB binding protein is known as a transcriptional co-activator that interacts with CREB and proteins in the basal transcriptional complex. Interestingly, CBP is required for EE-induced neurogenesis and cognitive enhancement [62]. While we observed a trend of increased levels of CaMKIV mRNA following EE, this trend did not reach statistical significance (Figure 6B). Another important mediator of learning and memory is the calcium-calmodulin-dependent protein kinase II (CaMKII) subunit. It regulates ion channel properties and synaptic trafficking of AMPA receptors during hippocampal LTP [63]–[65]. However, gene expression levels assayed by real time RT-PCR did not show any significant changes in CaMKII mRNA level (Figure 6C). Likewise, we did not observe upregulation of the postsynaptic density protein 95 (PSD-95), a well-characterized postsynaptic marker for plasticity, following experience in EE (Figure 6D).

Previously, we showed that EE upregulates hippocampal LTP [3], suggesting a role for EE in regulating synaptic plasticity. BDNF has been shown to increase mRNA expression level of members of the AMPA receptor family, glutamate receptor-1 (GluR1) and glutamate receptor-2 (GluR2) in hippocampal neurons, as well as mRNA and protein levels of ionotropic glutamate receptor, NMDA receptor subunits, NR1, NR2A and NR2B [66], [67]. By increasing the number of NMDA receptors, BNDF upregulates receptor activity and promotes LTP formation. To examine whether EE can induce expression of NMDA and AMPA receptors in APPSwe/PS1ΔE9 mice, we examined gene expression of NMDAR1 and GluR1 receptors following EE. We show that mRNA expression of NMDAR1 (Figure 6E), but not the AMPA receptor GluR1 (Figure 6F), was significantly induced following EE.

**Figure 7:**
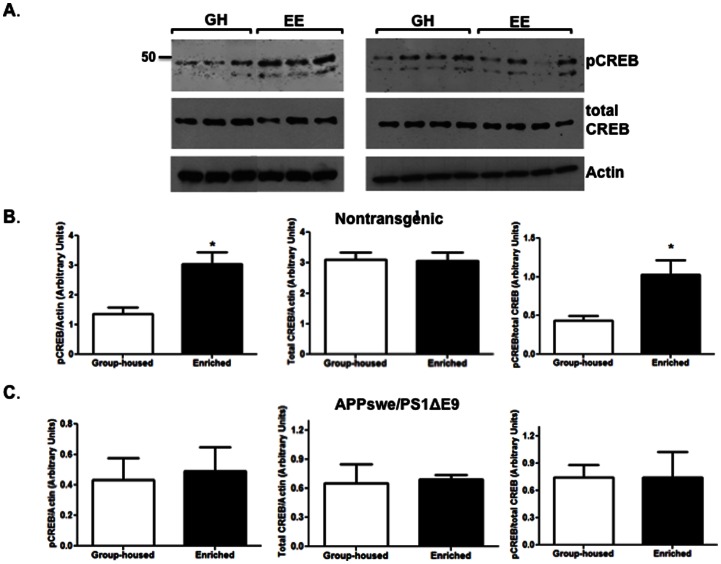
Environmental enrichment upregulates CREB phosphorylation in the hippocampus of wild type but not APPswe/PS1ΔE9 mice. (A) Western blot analysis of expression level of total CREB and phosphorylated CREB (pCREB) shows an increase in CREB phosphorylation in the hippocampus of wild type but not APPswe/PS1ΔE9 mice. Levels of total CREB were comparable in mice maintained in standard group- housing (GH) or EE in both genotypes. (B,C) Quantification of total CREB and pCREB levels in the hippocampus of (B) nontransgenic (*P<0.05 pCREB/actin, *P<0.05 pCREB/total CREB, *P* = 0.9161 total CREB/actin, N = 3 per group) and (C) APPswe/PS1ΔE9 mice (*P* = 0.8451 pCREB/actin, *P* = 0.9827 pCREB/total CREB, *P* = 0.8295 total CREB/actin, N = 4 per group).

**Figure 8:**
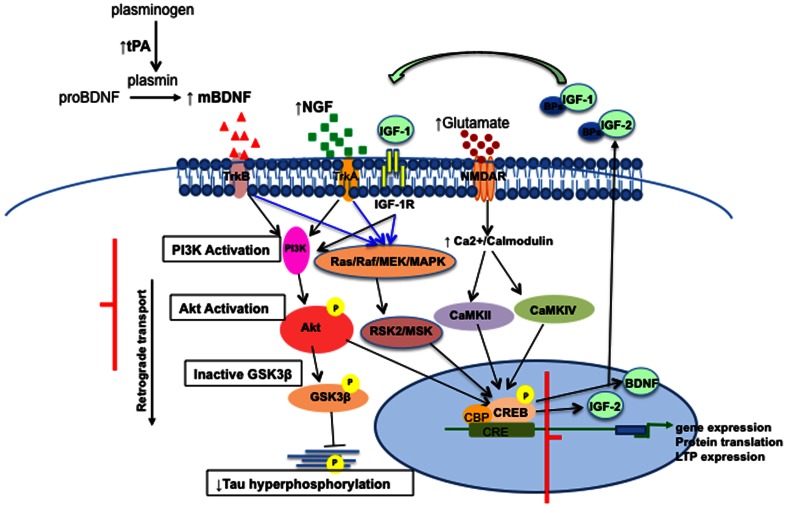
Proposed signaling network upregulated in the hippocampus of wild type and FAD-linked APPswe/PS1ΔE9 transgenic mice following environmental enrichment. Experience in environmental enrichment upregulates the expression of several neurotrophic factors, i.e., brain derived neurotrophic factor (BDNF) and nerve growth factor (NGF). Upregulation of insulin growth factor 1 (IGF-1) is observed in the hippocampus of APPswe/PS1ΔE9 but not wild type mice, while upregulation of neurotrophin-3 (NT-3) is observed in the hippocampi of wild type but not APPswe/PS1ΔE9 mice. Upregulation of tissue plasminogen activator (tPA) suggests that upregulation of BDNF signaling may be enhanced by the conversion of immature BDNF into the mature form by plasmin. Based on the observed upregulation of the BDNF receptor tyrosine kinase B (TrkB), we hypothesize that neurotrophin binding to their receptors activates intracellular signaling cascades. Intracellularly, several pathways are activated: Protein kinase B (Akt) is phosphorylated, and may downregulate glycogen synthase kinase β (GSK3β) activity that in turn, results in downregulation of tau phosphorylation. This pathway is blocked in the APPswe/PS1ΔE9 mice. Alternatively, a Mitogen-activated protein kinase pathway Ras/Raf/MEK/MAPK, 40S ribosomal protein S6 kinase and mitogen- and stress-activated protein kinase (RSK2/MSK) signaling might be activated following neurotrophin activation. In addition, neurotransmitter (i.e., NMDAR)-induced Ca^2+^/calmodulin-dependent protein kinase cascade is activated. These pathways lead to upregulation of CREB phosphorylation in wild type mice, which in turn, regulates gene expression necessary for the formation of long-term memory, including BDNF and IGF-2.

To examine the functional manifestation of increased CREB transcript following EE, we examined levels of total CREB and phosphorylated CREB at serine 133 (pCREB). Following neuronal stimulation, phosphorylation of CREB at serine 133 induces gene transcription that plays a crucial role in the initiation of learning and memory [68]. Western blot analysis shows that levels of total CREB are comparable in mice that experience either standard housing or EE (Figure 7A–C). Interestingly, there was an increase in levels of pCREB in the hippocampus of wild type mice following EE (Figure 7A,B), but not in the hippocampus of APPswe/PS1ΔE9 mice (Figure 7A,C), suggesting a defect in the signaling cascade initiating learning and memory. In summary, we propose a scheme that outlines the network of signaling pathways involved in EE-induced hippocampal plasticity as obtained in this study (Figure 8).

## Discussion

This study provides several important insights concerning the molecular mechanism underlying EE in wild type and APPswe/PS1ΔE9 mice. First, we show that EE modulates Akt and GSK3β activities in wild type mice. In contrast, these changes do not take place in APPswe/PS1ΔE9 mice, suggesting that APPswe/PS1ΔE9 mutations impair context-induced activation of Akt signaling and suppression of GSK3β activity. While it is plausible that EE enhances learning and memory and hippocampal plasticity in APPswe/PS1ΔE9 mice via one of the many other signaling pathways activated following EE, there appears to be a blockade of the Akt activation/GSK3β suppression pathway. GSK3β activity is altered in AD patients and in many AD mouse models [42], [69], [70], and possibly with aging. Thus, the lack of Akt activation and/or GSK3β suppression may have progressively detrimental effects on hippocampal plasticity, resulting in abnormal tau phosphorylation and inhibition of fast axonal transport. Further studies are warranted in order to determine whether impairments in the Akt pathway following EE in APPswe/PS1ΔE9 mice result of lack of activation or constitutive activation of this pathway.

Second, we show that EE upregulates several neurotrophic factors, including BDNF, NGF, IGF and NT-3. While an increase in BDNF has been observed previously in several FAD mouse models following EE, it wasn't clear whether other neurotrophic factors were being modulated as well. Increased levels in NGF [24], [54], [71], [72] and NT-3 [57], [58] were observed following EE in wild type rat brain. Expression of both BDNF and NGF is upregulated following EE in both wild type and APPswe/PS1ΔE9 mice, but NT-3 and IGF-1 are differentially regulated by EE in wild type and FAD mice. Interestingly, an increase in NT-3 gene expression level following EE occurs only in wild type mice, suggesting that APPswe/PS1ΔE9 mutants impair NT-3 signaling. In contrast, gene expression of IGF-1 was upregulated in the APPswe/PS1ΔE9, but not in wild type mice. Insulin growth factor-1 is implicated in promoting cell survival signaling pathways following environmental enrichment, and in regulation of neurogenesis through its survival promoting capacity [73], [74]. Activation of IGF-1 via its receptors promotes multiple molecular cascades including the PI3K/Akt pathway and the c-Src non-receptor tyrosine kinase, thereby modulating cell proliferation and cellular metabolism. Activity of IGF-1 receptor also appears to play a role in BDNF-mediated effects of physical activity on brain function. For example, IGF-1 signaling via IGF-1 receptor is necessary for exercise-induced upregulation of BDNF [55]. Increased IGF-1 production following exercise training may interact with BDNF to modulate synaptic plasticity, but the nature of functional overlap between the exercise-induced regulation of BDNF and IGF-1 has yet to be determined. Wolf and colleagues (2009) reported a different spectrum of changes in neurotrophin activity, with an increase in NT-3 and BDNF, but not in NGF or IGF in APP23 mice following EE. Such variations may be due to differences in transgene expression, genetic background, age of animals and duration of EE period [4]. Notably, the studies by Wolf and colleagues were examined in 17 month-old APP23 mice that experienced an EE for 11 months, whereas in our studies young mice were used, and their EE experience was for 1 month.

Third, we show that increased BDNF levels are accompanied by increased mRNA levels of TrkB receptors. It has been shown that expression levels of TrkB are decreased in the frontal cortex and hippocampal formation in AD, while the truncated form of TrkB is increased in association with decreases in BDNF mRNA levels in these brain regions [31], [75]. It is also suggested that decreased BDNF and TrkB expression may take place as a function of age or progression of the disease. We observed that BDNF and TrkB expression levels are comparable in wild type and FAD early in life, and that both receptor and ligand levels are upregulated following EE. Importantly, our data suggests that this increase is confined to the hippocampus, as no increase was observed in other cortical regions of these mice. Interestingly, we observed increased expression of tPA in both wild type and FAD hippocampus, suggesting that the increased level of BDNF may result from enhanced production of mature BDNF. Our data further suggest that enhanced BDNF/TrkB signaling following EE does not result in activation of the downstream signals, ERK and PLCγ/PKC pathways [19], suggesting that these pathways may not play a role in EE under the conditions examined in this study.

Fourth, we show that the expression of NMDA receptors, critical players in learning and memory, are significantly increased in the hippocampus of both wild type and APPswe/PS1ΔE9 mice following EE. The function of NMDA receptors is significantly reduced in AD patients, possibly contributing to memory deficits [76]. The increase in levels for the NMDA receptor subunits, NR1, NR2A and NR2B may be induced by BDNF [66], [67]. Our findings are consistent with Andin and colleagues who showed by *in situ* hybridization that EE upregulates NMDA mRNA expression, while the expression of AMPA mRNA is unchanged in the rat hippocampus [77]. Importantly, BDNF-mediated TrkB activation promotes neuronal synaptic activity via the activation of the CREB transcription factor, which drives expression and activation of intracellular signaling pathways through the action of AMPA and NDMA receptors [21], [78]. In support of that mechanism, we show that CREB transcription is upregulated following EE in both wild type and APPswe/PS1ΔE9 mice. A previous study reported that EE induces hippocampal level of CaMKII and CREB, but not ERK in neurogranin knockout mice [79]. While we show that mRNA levels of CREB are induced following EE, we do not observe an increase in total CREB levels. Nevertheless, we show for the first time that EE upregulates CREB phosphorylation in wild type mice, a critical process in the formation of long-term memory. In addition, we show that CREB phosphorylation following EE is impaired in the APPswe/PS1ΔE9 mice. Dysregulation of CREB has been implicated in a number of neurodegenerative diseases, including Alzheimer's disease [80]. Several studies suggest dysregulation of CREB, most of which has been attributed to elevated levels of Aβ [80]–[84]. Our observations are in agreement with Caccamo and colleagues suggesting that CREB phosphorylation is impaired following Morris Water Maze (MWM) in the 3XTg-AD mice [84]. Previous studies suggest that experience of mice in EE rescues learning and memory deficits in the MWM task in APPswe/PS1ΔE9 mice [85]. However, it is possible that other aspects of learning and memory not reflected in the MWM, cannot be rescued by EE.

To further elucidate molecular signaling pathways for CREB activation, we examined the expression of CaMKIV, an important Ca^2+^-activated CREB kinase *in vivo* that modifies CREB binding protein (CBP), and CaMKII. The activation via AMPA receptors and autophosphorylation of CaMKII has been shown to be critical for LTP formation [86]. However, we found no significant increase in levels of these kinases following EE. Further experiments are warranted for the understanding of the role of CREB signaling following EE, its dysfunction in AD and the implications of this dysfunction for learning and memory in these mice.

This study may suggest that an additive therapy aimed at regulating GSK3β, Akt and pCREB, critical players in multiple cellular processes and in learning and memory, might be necessary in order to reverse deficits that result from dysfunction of these pathways in AD. The functional implications of the lack of induction of CREB phosphorylation following EE in the APPswe/PS1ΔE9 mice is yet to be determined. In that regard, whether experience of FAD mice in EE fully rescues cognitive deficits is controversial and inconclusive [4], [85], [87], [88]. Several reports suggest that experience of FAD-linked APPswe/PS1ΔE9 and APP23 mice in EE rescues the performance of mice in the Morris Water Maze and the performance of PS1/PDAPP mice in the platform recognition and radial arm water maze [4], [85]. However, other studies report that EE could not rescue deficits in the Barnes maze tests and object recognition tasks, both of which are hippocampus-dependent tasks in TgCRND8 mice [87]. Levi and colleagues report that EE cannot elicit enhancement in learning and synaptic plasticity in apolipoprotein E4 (ApoE4)-expressing mice [88]. Importantly, whether Morris Water Maze and other learning and memory tests used in mice faithfully reflect all aspects of memory and cognition affected in AD is highly questionable. Thus, we claim that while EE has many beneficial effects on wild type and AD mouse brains, there might be aspects that are not adequately or sufficiently rescued.

In summary, this study sheds new light on the complex network of signaling pathways upregulated following EE in the brains of wild type and FAD- APPswe/PS1ΔE9 mice, and suggests that EE induces multiple molecular pathways, some of which are impaired in APPswe/PS1ΔE9 mice. This may suggest that translational approaches of EE, such as exercise, may be required but insufficient for the correction of learning and memory impairments in AD.

## Supporting Information

Table S1 **Primer sequences used for real time RT-PCR.**
(DOC)

## References

[1] Lazarov O, Larson J (2007) Environmental enrichment: from mouse AD model to AD therapy; Sun M, editor: Nova Science Publishers, Inc.

[2] Lazarov O, Robinson J, Tang YP, Hairston IS, Korade-Mirnics Z, et al (2005) Environmental enrichment reduces Abeta levels and amyloid deposition in transgenic mice. Cell 120: 701–713. 15766532 10.1016/j.cell.2005.01.015

[3] Hu YS, Xu P, Pigino G, Brady ST, Larson J, et al (2010) Complex environment experience rescues impaired neurogenesis, enhances synaptic plasticity, and attenuates neuropathology in familial Alzheimer's disease-linked APPswe/PS1{Delta}E9 mice. Faseb J 24: 1667–1681. 20086049 10.1096/fj.09-136945 PMC4050966

[4] Wolf SA, Kronenberg G, Lehmann K, Blankenship A, Overall R, et al (2006) Cognitive and physical activity differently modulate disease progression in the amyloid precursor protein (APP)-23 model of Alzheimer's disease. Biol Psychiatry 60: 1314–1323. 16806094 10.1016/j.biopsych.2006.04.004

[5] Lazarov O, Morfini GA, Lee EB, Farah MH, Szodorai A, et al (2005) Axonal transport, amyloid precursor protein, kinesin-1, and the processing apparatus: revisited. J Neurosci 25: 2386–2395. 15745965 10.1523/JNEUROSCI.3089-04.2005 PMC6726084

[6] Adlard PA, Perreau VM, Pop V, Cotman CW (2005) Voluntary exercise decreases amyloid load in a transgenic model of Alzheimer's disease. J Neurosci 25: 4217–4221. 15858047 10.1523/JNEUROSCI.0496-05.2005 PMC6725122

[7] Billings LM, Green KN, McGaugh JL, LaFerla FM (2007) Learning decreases Abeta*56 and tau pathology and ameliorates behavioral decline in 3xTg-AD mice. J Neurosci 27: 751–761. 17251414 10.1523/JNEUROSCI.4800-06.2007 PMC6672918

[8] Chen MJ, Russo-Neustadt AA (2005) Exercise activates the phosphatidylinositol 3-kinase pathway. Brain Res Mol Brain Res 135: 181–193. 15857681 10.1016/j.molbrainres.2004.12.001

[9] Sakamoto K, Arnolds DE, Ekberg I, Thorell A, Goodyear LJ (2004) Exercise regulates Akt and glycogen synthase kinase-3 activities in human skeletal muscle. Biochem Biophys Res Commun 319: 419–425. 15178423 10.1016/j.bbrc.2004.05.020

[10] Bruel-Jungerman E, Veyrac A, Dufour F, Horwood J, Laroche S, et al (2009) Inhibition of PI3K-Akt signaling blocks exercise-mediated enhancement of adult neurogenesis and synaptic plasticity in the dentate gyrus. PLoS One 4: e7901. 19936256 10.1371/journal.pone.0007901 PMC2775944

[11] Hernandez F, Lucas JJ, Avila J (2012) GSK3 and Tau: Two Convergence Points in Alzheimer's Disease. J Alzheimers Dis 10.3233/JAD-2012-129025 22710914

[12] Lopez-Tobon A, Castro-Alvarez JF, Piedrahita D, Boudreau RL, Gallego-Gomez JC, et al (2011) Silencing of CDK5 as potential therapy for Alzheimer's disease. Rev Neurosci 22: 143–152. 21476938 10.1515/RNS.2011.015

[13] Ferrer I, Gomez-Isla T, Puig B, Freixes M, Ribe E, et al (2005) Current advances on different kinases involved in tau phosphorylation, and implications in Alzheimer's disease and tauopathies. Curr Alzheimer Res 2: 3–18. 15977985 10.2174/1567205052772713

[14] Avila J, Wandosell F, Hernandez F (2010) Role of glycogen synthase kinase-3 in Alzheimer's disease pathogenesis and glycogen synthase kinase-3 inhibitors. Expert Rev Neurother 10: 703–710. 20420491 10.1586/ern.10.40

[15] Baum L, Hansen L, Masliah E, Saitoh T (1996) Glycogen synthase kinase 3 alteration in Alzheimer disease is related to neurofibrillary tangle formation. Mol Chem Neuropathol 29: 253–261. 8971700 10.1007/BF02815006

[16] Kaytor MD, Orr HT (2002) The GSK3 beta signaling cascade and neurodegenerative disease. Current opinion in neurobiology 12: 275–278. 12049933 10.1016/s0959-4388(02)00320-3

[17] Morfini G, Szebenyi G, Elluru R, Ratner N, Brady ST (2002) Glycogen synthase kinase 3 phosphorylates kinesin light chains and negatively regulates kinesin-based motility. Embo J 21: 281–293. 11823421 10.1093/emboj/21.3.281 PMC125832

[18] Morfini G, Szebenyi G, Brown H, Pant HC, Pigino G, et al (2004) A novel CDK5-dependent pathway for regulating GSK3 activity and kinesin-driven motility in neurons. Embo J 23: 2235–2245. 15152189 10.1038/sj.emboj.7600237 PMC419914

[19] Ohira K, Hayashi M (2009) A new aspect of the TrkB signaling pathway in neural plasticity. Curr Neuropharmacol 7: 276–285. 20514207 10.2174/157015909790031210 PMC2811861

[20] Sakamoto K, Karelina K, Obrietan K (2011) CREB: a multifaceted regulator of neuronal plasticity and protection. J Neurochem 116: 1–9. 21044077 10.1111/j.1471-4159.2010.07080.x PMC3575743

[21] Shieh PB, Ghosh A (1999) Molecular mechanisms underlying activity-dependent regulation of BDNF expression. Journal of neurobiology 41: 127–134. 10504200

[22] Adlard PA, Cotman CW (2004) Voluntary exercise protects against stress-induced decreases in brain-derived neurotrophic factor protein expression. Neuroscience 124: 985–992. 15026138 10.1016/j.neuroscience.2003.12.039

[23] Adlard PA, Perreau VM, Cotman CW (2005) The exercise-induced expression of BDNF within the hippocampus varies across life-span. Neurobiol Aging 26: 511–520. 15653179 10.1016/j.neurobiolaging.2004.05.006

[24] Pham TM, Ickes B, Albeck D, Soderstrom S, Granholm AC, et al (1999) Changes in brain nerve growth factor levels and nerve growth factor receptors in rats exposed to environmental enrichment for one year. Neuroscience 94: 279–286. 10613518 10.1016/s0306-4522(99)00316-4

[25] Nithianantharajah J, Hannan AJ (2006) Enriched environments, experience-dependent plasticity and disorders of the nervous system. Nat Rev Neurosci 7: 697–709. 16924259 10.1038/nrn1970

[26] Murer MG, Yan Q, Raisman-Vozari R (2001) Brain-derived neurotrophic factor in the control human brain, and in Alzheimer's disease and Parkinson's disease. Prog Neurobiol 63: 71–124. 11040419 10.1016/s0301-0082(00)00014-9

[27] Phillips HS, Hains JM, Armanini M, Laramee GR, Johnson SA, et al (1991) BDNF mRNA is decreased in the hippocampus of individuals with Alzheimer's disease. Neuron 7: 695–702. 1742020 10.1016/0896-6273(91)90273-3

[28] Narisawa-Saito M, Nawa H (1996) Differential regulation of hippocampal neurotrophins during aging in rats. J Neurochem 67: 1124–1131. 8752119 10.1046/j.1471-4159.1996.67031124.x

[29] Narisawa-Saito M, Wakabayashi K, Tsuji S, Takahashi H, Nawa H (1996) Regional specificity of alterations in NGF, BDNF and NT-3 levels in Alzheimer's disease. Neuroreport 7: 2925–2928. 9116211 10.1097/00001756-199611250-00024

[30] Murer MG, Boissiere F, Yan Q, Hunot S, Villares J, et al (1999) An immunohistochemical study of the distribution of brain-derived neurotrophic factor in the adult human brain, with particular reference to Alzheimer's disease. Neuroscience 88: 1015–1032. 10336117 10.1016/s0306-4522(98)00219-x

[31] Ferrer I, Marin C, Rey MJ, Ribalta T, Goutan E, et al (1999) BDNF and full-length and truncated TrkB expression in Alzheimer disease. Implications in therapeutic strategies. J Neuropathol Exp Neurol 58: 729–739. 10411343 10.1097/00005072-199907000-00007

[32] Durany N, Michel T, Kurt J, Cruz-Sanchez FF, Cervas-Navarro J, et al (2000) Brain-derived neurotrophic factor and neurotrophin-3 levels in Alzheimer's disease brains. Int J Dev Neurosci 18: 807–813. 11156744

[33] Michalski B, Fahnestock M (2003) Pro-brain-derived neurotrophic factor is decreased in parietal cortex in Alzheimer's disease. Brain Res Mol Brain Res 111: 148–154. 12654514 10.1016/s0169-328x(03)00003-2

[34] Hock C, Heese K, Hulette C, Rosenberg C, Otten U (2000) Region-specific neurotrophin imbalances in Alzheimer disease: decreased levels of brain-derived neurotrophic factor and increased levels of nerve growth factor in hippocampus and cortical areas. Arch Neurol 57: 846–851. 10867782 10.1001/archneur.57.6.846

[35] Elliott E, Atlas R, Lange A, Ginzburg I (2005) Brain-derived neurotrophic factor induces a rapid dephosphorylation of tau protein through a PI-3 Kinase signalling mechanism. Eur J Neurosci 22: 1081–1089. 16176349 10.1111/j.1460-9568.2005.04290.x

[36] Jankowsky JL, Slunt HH, Ratovitski T, Jenkins NA, Copeland NG, et al (2001) Co-expression of multiple transgenes in mouse CNS: a comparison of strategies. Biomol Eng 17: 157–165. 11337275 10.1016/s1389-0344(01)00067-3

[37] Demars M, Hu YS, Gadadhar A, Lazarov O (2010) Impaired neurogenesis is an early event in the etiology of familial Alzheimer's disease in transgenic mice. Journal of neuroscience research 88: 2103–2117. 20209626 10.1002/jnr.22387 PMC3696038

[38] Szapacs ME, Mathews TA, Tessarollo L, Ernest Lyons W, Mamounas LA, et al (2004) Exploring the relationship between serotonin and brain-derived neurotrophic factor: analysis of BDNF protein and extraneuronal 5-HT in mice with reduced serotonin transporter or BDNF expression. J Neurosci Methods 140: 81–92. 15589338 10.1016/j.jneumeth.2004.03.026

[39] Livak KJ, Schmittgen TD (2001) Analysis of relative gene expression data using real-time quantitative PCR and the 2(-Delta Delta C(T)) Method. Methods 25: 402–408. 11846609 10.1006/meth.2001.1262

[40] Ryder J, Su Y, Ni B (2004) Akt/GSK3beta serine/threonine kinases: evidence for a signalling pathway mediated by familial Alzheimer's disease mutations. Cell Signal 16: 187–200. 14636889 10.1016/j.cellsig.2003.07.004

[41] Hernandez P, Lee G, Sjoberg M, Maccioni RB (2009) Tau phosphorylation by cdk5 and Fyn in response to amyloid peptide Abeta (25–35): involvement of lipid rafts. J Alzheimers Dis 16: 149–156. 19158430 10.3233/JAD-2009-0933

[42] Hernandez F, Gomez de Barreda E, Fuster-Matanzo A, Lucas JJ, Avila J (2010) GSK3: a possible link between beta amyloid peptide and tau protein. Exp Neurol 223: 322–325. 19782073 10.1016/j.expneurol.2009.09.011

[43] Blalock EM, Geddes JW, Chen KC, Porter NM, Markesbery WR, et al (2004) Incipient Alzheimer's disease: microarray correlation analyses reveal major transcriptional and tumor suppressor responses. Proc Natl Acad Sci U S A 101: 2173–2178. 14769913 10.1073/pnas.0308512100 PMC357071

[44] DaRocha-Souto B, Coma M, Perez-Nievas BG, Scotton TC, Siao M, et al (2012) Activation of glycogen synthase kinase-3 beta mediates beta-amyloid induced neuritic damage in Alzheimer's disease. Neurobiol Dis 45: 425–437. 21945540 10.1016/j.nbd.2011.09.002 PMC3694284

[45] Leroy K, Yilmaz Z, Brion JP (2007) Increased level of active GSK-3beta in Alzheimer's disease and accumulation in argyrophilic grains and in neurones at different stages of neurofibrillary degeneration. Neuropathol Appl Neurobiol 33: 43–55. 17239007 10.1111/j.1365-2990.2006.00795.x

[46] Pei JJ, Tanaka T, Tung YC, Braak E, Iqbal K, et al (1997) Distribution, levels, and activity of glycogen synthase kinase-3 in the Alzheimer disease brain. J Neuropathol Exp Neurol 56: 70–78. 8990130 10.1097/00005072-199701000-00007

[47] Hye A, Kerr F, Archer N, Foy C, Poppe M, et al (2005) Glycogen synthase kinase-3 is increased in white cells early in Alzheimer's disease. Neurosci Lett 373: 1–4. 15555766 10.1016/j.neulet.2004.10.031

[48] Frame S, Cohen P, Biondi RM (2001) A common phosphate binding site explains the unique substrate specificity of GSK3 and its inactivation by phosphorylation. Mol Cell 7: 1321–1327. 11430833 10.1016/s1097-2765(01)00253-2

[49] Numakawa T, Adachi N, Richards M, Chiba S, Kunugi H (2012) Brain-Derived Neurotrophic Factor and Glucocorticoids: Reciprocal Influence in the central nervous system. Neuroscience 10.1016/j.neuroscience.2012.09.073 23069755

[50] Angelucci F, Spalletta G, di Iulio F, Ciaramella A, Salani F, et al (2010) Alzheimer's disease (AD) and Mild Cognitive Impairment (MCI) patients are characterized by increased BDNF serum levels. Curr Alzheimer Res 7: 15–20. 20205668 10.2174/156720510790274473

[51] Hock C, Heese K, Muller-Spahn F, Hulette C, Rosenberg C, et al (1998) Decreased trkA neurotrophin receptor expression in the parietal cortex of patients with Alzheimer's disease. Neurosci Lett 241: 151–154. 9507943 10.1016/s0304-3940(98)00019-6

[52] Ding Q, Ying Z, Gomez-Pinilla F (2011) Exercise influences hippocampal plasticity by modulating brain-derived neurotrophic factor processing. Neuroscience 192: 773–780. 21756980 10.1016/j.neuroscience.2011.06.032 PMC3225196

[53] Sartori CR, Vieira AS, Ferrari EM, Langone F, Tongiorgi E, et al (2011) The antidepressive effect of the physical exercise correlates with increased levels of mature BDNF, and proBDNF proteolytic cleavage-related genes, p11 and tPA. Neuroscience 180: 9–18. 21371535 10.1016/j.neuroscience.2011.02.055

[54] Pham TM, Winblad B, Granholm AC, Mohammed AH (2002) Environmental influences on brain neurotrophins in rats. Pharmacology, biochemistry, and behavior 73: 167–175. 10.1016/s0091-3057(02)00783-9 12076736

[55] Ding Q, Vaynman S, Akhavan M, Ying Z, Gomez-Pinilla F (2006) Insulin-like growth factor I interfaces with brain-derived neurotrophic factor-mediated synaptic plasticity to modulate aspects of exercise-induced cognitive function. Neuroscience 140: 823–833. 16650607 10.1016/j.neuroscience.2006.02.084

[56] Ickes BR, Pham TM, Sanders LA, Albeck DS, Mohammed AH, et al (2000) Long-term environmental enrichment leads to regional increases in neurotrophin levels in rat brain. Exp Neurol 164: 45–52. 10877914 10.1006/exnr.2000.7415

[57] Torasdotter M, Metsis M, Henriksson BG, Winblad B, Mohammed AH (1996) Expression of neurotrophin-3 mRNA in the rat visual cortex and hippocampus is influenced by environmental conditions. Neurosci Lett 218: 107–110. 8945739 10.1016/s0304-3940(96)13127-x

[58] Torasdotter M, Metsis M, Henriksson BG, Winblad B, Mohammed AH (1998) Environmental enrichment results in higher levels of nerve growth factor mRNA in the rat visual cortex and hippocampus. Behav Brain Res 93: 83–90. 9659990 10.1016/s0166-4328(97)00142-3

[59] Marie H, Morishita W, Yu X, Calakos N, Malenka RC (2005) Generation of silent synapses by acute in vivo expression of CaMKIV and CREB. Neuron 45: 741–752. 15748849 10.1016/j.neuron.2005.01.039

[60] Kang H, Sun LD, Atkins CM, Soderling TR, Wilson MA, et al (2001) An important role of neural activity-dependent CaMKIV signaling in the consolidation of long-term memory. Cell 106: 771–783. 11572782 10.1016/s0092-8674(01)00497-4

[61] Ho N, Liauw JA, Blaeser F, Wei F, Hanissian S, et al (2000) Impaired synaptic plasticity and cAMP response element-binding protein activation in Ca2+/calmodulin-dependent protein kinase type IV/Gr-deficient mice. J Neurosci 20: 6459–6472. 10964952 10.1523/JNEUROSCI.20-17-06459.2000 PMC6772951

[62] Lopez-Atalaya JP, Ciccarelli A, Viosca J, Valor LM, Jimenez-Minchan M, et al (2011) CBP is required for environmental enrichment-induced neurogenesis and cognitive enhancement. Embo J 30: 4287–4298. 21847097 10.1038/emboj.2011.299 PMC3199387

[63] Barria A, Muller D, Derkach V, Griffith LC, Soderling TR (1997) Regulatory phosphorylation of AMPA-type glutamate receptors by CaM-KII during long-term potentiation. Science 276: 2042–2045. 9197267 10.1126/science.276.5321.2042

[64] Derkach V, Barria A, Soderling TR (1999) Ca2+/calmodulin-kinase II enhances channel conductance of alpha-amino-3-hydroxy-5-methyl-4-isoxazolepropionate type glutamate receptors. Proc Natl Acad Sci U S A 96: 3269–3274. 10077673 10.1073/pnas.96.6.3269 PMC15931

[65] Esteban JA, Shi SH, Wilson C, Nuriya M, Huganir RL, et al (2003) PKA phosphorylation of AMPA receptor subunits controls synaptic trafficking underlying plasticity. Nat Neurosci 6: 136–143. 12536214 10.1038/nn997

[66] Caldeira MV, Melo CV, Pereira DB, Carvalho RF, Carvalho AL, et al (2007) BDNF regulates the expression and traffic of NMDA receptors in cultured hippocampal neurons. Mol Cell Neurosci 35: 208–219. 17428676 10.1016/j.mcn.2007.02.019

[67] Caldeira MV, Melo CV, Pereira DB, Carvalho R, Correia SS, et al (2007) Brain-derived neurotrophic factor regulates the expression and synaptic delivery of alpha-amino-3-hydroxy-5-methyl-4-isoxazole propionic acid receptor subunits in hippocampal neurons. J Biol Chem 282: 12619–12628. 17337442 10.1074/jbc.M700607200

[68] Lee YS, Silva AJ (2009) The molecular and cellular biology of enhanced cognition. Nat Rev Neurosci 10: 126–140. 19153576 10.1038/nrn2572 PMC2664745

[69] Takashima A, Murayama M, Murayama O, Kohno T, Honda T, et al (1998) Presenilin 1 associates with glycogen synthase kinase-3beta and its substrate tau. Proc Natl Acad Sci U S A 95: 9637–9641. 9689133 10.1073/pnas.95.16.9637 PMC21391

[70] Hernandez F, de Barreda EG, Fuster-Matanzo A, Goni-Oliver P, Lucas JJ, et al (2009) The role of GSK3 in Alzheimer disease. Brain Res Bull 80: 248–250. 19477245 10.1016/j.brainresbull.2009.05.017

[71] Olsson T, Mohammed AH, Donaldson LF, Henriksson BG, Seckl JR (1994) Glucocorticoid receptor and NGFI-A gene expression are induced in the hippocampus after environmental enrichment in adult rats. Brain Res Mol Brain Res 23: 349–353. 8090075 10.1016/0169-328x(94)90246-1

[72] Pham TM, Soderstrom S, Winblad B, Mohammed AH (1999) Effects of environmental enrichment on cognitive function and hippocampal NGF in the non-handled rats. Behavioural brain research 103: 63–70. 10475165 10.1016/s0166-4328(99)00019-4

[73] Aberg MA, Aberg ND, Hedbacker H, Oscarsson J, Eriksson PS (2000) Peripheral infusion of IGF-I selectively induces neurogenesis in the adult rat hippocampus. J Neurosci 20: 2896–2903. 10751442 10.1523/JNEUROSCI.20-08-02896.2000 PMC6772218

[74] Lichtenwalner RJ, Forbes ME, Sonntag WE, Riddle DR (2006) Adult-onset deficiency in growth hormone and insulin-like growth factor-I decreases survival of dentate granule neurons: insights into the regulation of adult hippocampal neurogenesis. Journal of neuroscience research 83: 199–210. 16385581 10.1002/jnr.20719

[75] Connor B, Young D, Yan Q, Faull RL, Synek B, et al (1997) Brain-derived neurotrophic factor is reduced in Alzheimer's disease. Brain Res Mol Brain Res 49: 71–81. 9387865 10.1016/s0169-328x(97)00125-3

[76] Greenamyre JT, Penney JB, D'Amato CJ, Young AB (1987) Dementia of the Alzheimer's type: changes in hippocampal L-[3H]glutamate binding. J Neurochem 48: 543–551. 2878980 10.1111/j.1471-4159.1987.tb04127.x

[77] Andin J, Hallbeck M, Mohammed AH, Marcusson J (2007) Influence of environmental enrichment on steady-state mRNA levels for EAAC1, AMPA1 and NMDA2A receptor subunits in rat hippocampus. Brain Res 1174: 18–27. 17854777 10.1016/j.brainres.2007.06.101

[78] Lau GC, Saha S, Faris R, Russek SJ (2004) Up-regulation of NMDAR1 subunit gene expression in cortical neurons via a PKA-dependent pathway. J Neurochem 88: 564–575. 14720206 10.1046/j.1471-4159.2003.02156.x

[79] Huang FL, Huang KP, Wu J, Boucheron C (2006) Environmental enrichment enhances neurogranin expression and hippocampal learning and memory but fails to rescue the impairments of neurogranin null mutant mice. J Neurosci 26: 6230–6237. 16763030 10.1523/JNEUROSCI.1182-06.2006 PMC6675199

[80] Ma QL, Harris-White ME, Ubeda OJ, Simmons M, Beech W, et al (2007) Evidence of Abeta- and transgene-dependent defects in ERK-CREB signaling in Alzheimer's models. J Neurochem 103: 1594–1607. 17760871 10.1111/j.1471-4159.2007.04869.x PMC2527620

[81] Dineley KT, Westerman M, Bui D, Bell K, Ashe KH, et al (2001) Beta-amyloid activates the mitogen-activated protein kinase cascade via hippocampal alpha7 nicotinic acetylcholine receptors: In vitro and in vivo mechanisms related to Alzheimer's disease. J Neurosci 21: 4125–4133. 11404397 10.1523/JNEUROSCI.21-12-04125.2001 PMC6762764

[82] Yamamoto-Sasaki M, Ozawa H, Saito T, Rosler M, Riederer P (1999) Impaired phosphorylation of cyclic AMP response element binding protein in the hippocampus of dementia of the Alzheimer type. Brain Res 824: 300–303. 10196463 10.1016/s0006-8993(99)01220-2

[83] Muller M, Cardenas C, Mei L, Cheung KH, Foskett JK (2011) Constitutive cAMP response element binding protein (CREB) activation by Alzheimer's disease presenilin-driven inositol trisphosphate receptor (InsP3R) Ca2+ signaling. Proc Natl Acad Sci U S A 108: 13293–13298. 21784978 10.1073/pnas.1109297108 PMC3156223

[84] Caccamo A, Maldonado MA, Bokov AF, Majumder S, Oddo S (2010) CBP gene transfer increases BDNF levels and ameliorates learning and memory deficits in a mouse model of Alzheimer's disease. Proc Natl Acad Sci U S A 107: 22687–22692. 21149712 10.1073/pnas.1012851108 PMC3012497

[85] Jankowsky JL, Melnikova T, Fadale DJ, Xu GM, Slunt HH, et al (2005) Environmental enrichment mitigates cognitive deficits in a mouse model of Alzheimer's disease. J Neurosci 25: 5217–5224. 15917461 10.1523/JNEUROSCI.5080-04.2005 PMC4440804

[86] Fukunaga K, Muller D, Miyamoto E (1996) CaM kinase II in long-term potentiation. Neurochem Int 28: 343–358. 8740440 10.1016/0197-0186(95)00097-6

[87] Gortz N, Lewejohann L, Tomm M, Ambree O, Keyvani K, et al (2008) Effects of environmental enrichment on exploration, anxiety, and memory in female TgCRND8 Alzheimer mice. Behav Brain Res 191: 43–48. 18433890 10.1016/j.bbr.2008.03.006

[88] Levi O, Jongen-Relo AL, Feldon J, Roses AD, Michaelson DM (2003) ApoE4 impairs hippocampal plasticity isoform-specifically and blocks the environmental stimulation of synaptogenesis and memory. Neurobiol Dis 13: 273–282. 12901842 10.1016/s0969-9961(03)00045-7

