# Supplementary material for: Molecular Mechanisms of Environmental Enrichment: Impairments in Akt/GSK3β, Neurotrophin-3 and CREB Signaling
Source: PLoS One. 2013 May 21;8(5):e64460. doi: 10.1371/journal.pone.0064460 (PMC3660250; doi:10.1371/journal.pone.0064460)
Supplement: Table S1 — Primer sequences used for real time RT-PCR. (DOC) [file pone.0064460.s001.doc]

**Supplementary data**

Table 1: Primer sequences used for real time RT-PCR.

| **Targets** | **Sequences** | **References** |
| --- | --- | --- |
| BDNF | Forward 5’-CCA TAA GGA CGC GGA CTT GT-3’  Reverse 5’-GAG GCT CCA AAG GCA CTT GA-3’ | [89] |
| CaMKIIα | Forward 5’-ACG AGG GAG TAG ACA GTG GAA GAC-3’  Reverse 5’-GCA GGC AGC AGA GTA GTG GAC-3’ | [90] |
| CaMKIV | Forward 5’-AAA TCA GCC TGG TCC TTG AG-3’  Reverse 5’-TCT GGT TTG AGG TCA CGA TG-3’ | [91] |
| CBP | Forward 5’-CAC AGG CAG GAG GCA TGA C-3’  Reverse 5’-CAG TTT GAC TAA AGG GTT GTC CAA-3’ | [62] |
| CREB | Forward 5’-GGA ATC TGG AGC AGA CAA CC-3’  Reverse 5’-ATA ACG CCA TGG ACC TGG AC-3’ | [91] |
| GluR1 | Forward 5’-GCT TTG TCA CAA CTC ACG GA-3′  Reverse 5′ CCT TTG GAG AAC TGG GAA CA-3’ | [62,92] |
| IGF-1 | Forward 5’-CTG GGC TAG GAA CTG TGA GC-3’  Reverse 5’-TAA GTG CCG TAT CCC AGA GG-3’ | [4] |
| NGF | Forward 5’-CAG GCA GAA CCG TAC ACA GA-3’  Reverse 5’-GTC TGA AGA GGT GGG TGG AG-3’ | [4] |
| NMDAR | Forward 5'- AAG AGG AGT TCA CAG TCA ATG G -3'  Reverse 5'- CAG GTC AAC GCA GAA GCC -3' | [90] |
| NT-3 | Forward 5’-TGC AAC GGA CAC AGA GCT AC-3’  Reverse 5’-TGC CCA CAT AAT CCT CCA TT-3’ | [4] |
| PKC-γ | Forward 5’-CTC GTT TCT TCA AGC AGC CAA-3’  Reverse 5’-GTG AAC CAC AAA GCT ACA GAC T-3’ | [93] |
| PSD-95 | Forward 5′-GGT GAC GAC CCA TCC ATC TTT ATC-3′  Reverse 5′-CGG ACA TCC ACT TCA TTG ACA AAC-3′ | [94] |
| tPA | Forward 5’-TGT CTT TAA GGC AGG GAA GT-3’  Reverse 5’-GTC ACA CCT TTC CCA ACA TA-3’ | [53] |
| TrkB | Forward 5-‘CGC CCT GTG AGC TGA ACT CTG-3’  Reverse 5’-CTG CTT CTC AGC TGC CTG ACC-3’ | [95] |
| **Targets** | **Internal Controls** | **References** |
| 18S rRNA | Forward 5’-CGG CTA CCA CAT CCA AGG AA-3’  Reverse 5’-GCT GGA ATT ACC GCG GCT-3’ | [96] |
| GAPDH | Forward 5’-TGG TCT ACA TGT TCC AGT ATG AC-3’  Reverse 5’-TAG ACT CCA CGA CAT ACT CAG C-3’ | [90] |

All primer sequences were published previously as indicated in the references.
